# Acceleration Mechanism of Steel Slag Hydration Using THEED

**DOI:** 10.3390/ma17040858

**Published:** 2024-02-12

**Authors:** Deyu Yue, Jianfeng Wang, Pengchen Huo, Lei Chang, Dingyong He, Suping Cui, Hui Liu

**Affiliations:** 1 College of Materials Science and Engineering, Beijing University of Technology, Beijing 100124, China; yuedy0511@139.com (D.Y.); wangjianfeng@bjut.edu.cn (J.W.); huopengchenbjut@126.com (P.H.); changlei0127@outlook.com (L.C.); dyhe@bjut.edu.cn (D.H.); cuisuping@bjut.edu.cn (S.C.); 2 Key Laboratory of Advanced Functional Materials, Ministry of Education, Beijing University of Technology, Beijing 100124, China

**Keywords:** steel slag, THEED, hydration, dissolution, chelation

## Abstract

In this paper, the strength development of a pure steel slag (SS) system with various concentrations of N,N,N′,N′-Tetrakis-(2-hydroxyethyl) ethylenediamine (THEED) was investigated. The hydration kinetics, pore structure and microstructure of SS pastes with and without THEED were characterized to underscore the working mechanism of THEED. Results show that THEED additions significantly increase the 3, 7 and 28 days compressive strength of hardened SS pastes. The enhancement effect increases with the dosage of THEED. At a concentration of 2000 ppm, THEED increased the compressive strength by 733%, 665%, and 545% at 3, 7 and 28 days, respectively. It is confirmed that THEED additions improve the hydration degree of SS by accelerating hydration of the aluminum phase (C_3_A, PDF-38-1429; C_12_A_7_, PDF-48-1882) and C_2_F,( PDF 38-0408) to generate Mc (PDF-41-0219) and Pa (PDF-30-0222) in the presence of CaCO_3_. Also, the hydration degree of silicates is increased by THEED. In this way, THEED additions refine the pore structure of hardened SS paste by increasing the pore volume with a diameter below 300 nm to achieve enhancement. The chelating effect of THEED results in promoting dissolution of SS, which provides the driving force for accelerating SS hydration.

## 1. Introduction

Steel slag (SS) is a by-product of steel manufacturing, accounting for 15–20% of crude steel production [1]. In China, the annual output of crude steel reached 1033 million tons in 2021, resulting in more than 150 million tons of SS being produced [2,3]. However, in recent years more than 70% of the SS remains to be utilized [4,5]. SS can be effectively used as a supplementary cementitious material. Unfortunately, the minimal hydraulic activity of SS is a key factor in limiting its utilization efficiency. Currently, methods of improving the hydraulic activity of SS include mechanical grinding [6]; acid activation [7]; alkali activation [8]; high-temperature curing [9]; tempering/reconstruction [10]; and organic activation methods [11].

Alkanolamines are widely used as admixtures of organic small molecules to regulate the hydration of cementitious materials. Commonly used alkanolamines include N,N,N′,N′-Tetrakis-(2-hydroxyethyl) ethylenediamine (THEED); triethanolamine (TEA); triisopropanolamine (TIPA); diethanol-isopropanolamine (DEIPA); and ethanol-diisopropanolamine (EDIPA) [12,13], which share a similar molecular structure. In cement pastes, alkanolamines such as TEA promote the aluminum phases (C_3_A and C_4_AF) of hydration to generate AFt (ettringite) [14], thus increasing the time for gypsum depletion. In this way, TEA triggers the rehydration of C_3_A and the transformation of AFt to AFm (monosulphoaluminate hydrate) [15]. However, TEA addition inhibits C_3_S hydration, especially at a high dosage, as confirmed by Lu et al. [16]. Due to the opposite effects on hydration of aluminum phases and silicate phases, trace alkanolamines (e.g., 0.03 wt%) can increase the early strength of cement mortars. At a higher dosage (e.g., 0.1 wt%), alkanolamines delay the strength development of cement mortars. After investigating the role of alkanolamines in cement slurry, it was found that TEA, DEIPA, TIPA and EDIPA exhibited a chelating ability with metal ions such as Ca^2+^, Al^3+^, and Fe^3+^ [17,18]. The effect was related to the dissolution of cement clinkers [19]. Therefore, alkanolamines have been developed as additives for increasing the hydraulic activity of SS. Such alkanolamines increase the hydration degree of SS or cement to enhance the compressive strength of composite mortars. The intensity of the second hydration peak in hydrating SS-cement paste was observed to be decreased by TEA, DEIPA, and MDEA, but increased by TIPA. Unfortunately, it is hard to determine the hydration processes of SS and cement separately in a SS–cement composite. Recently, in a pure SS paste with KOH, EDIPA additions were found to promote the hydration and carbonation of aluminate and ferrite phases, thus enhancing compressive strength. However, more details on SS hydration are needed to be investigated for further understanding of the acceleration mechanism of such alkanolamines.

THEED is typically used as a chelating agent in the field of electroless copper plating. In recent years, it has been employed as a cement additive for regulating the setting and hardening of cement pastes. Evidence shows that THEED improves the hydration degree of cement, and its compressive strength [20]. Xu et al. [21] found that 0.02 wt% and 0.04 wt% THEED improved the degree of cement hydration before 56 d. In a slag–cement system, 0.01–0.10 wt% THEED additions were found to improve strength before 28 d [22]. The pozzolanic-promoting reaction of titanium slag using THEED is proposed as the mechanism of strength enhancement. However, the hydration process of a pure SS system in the presence of THEED is not clear.

## 2. Experimental Section

### 2.1. Materials

The cementitious material was a hot-stewed steel slag (SS) with a surface area of 520 m^2^/kg, a by-product of the basic oxygen furnace steelmaking process, which was provided by Shandong Luli Steel Company Limited. The chemical and mineral composition of the SS determined using XRF and XRD is shown in Table 1 and Table 2. N,N,N′,N′-Tetrakis-(2-hydroxyethyl) ethylenediamine (THEED) with 99% purity was purchased from Shanghai Macklin Biochemical Co., Ltd. (Shanghai, China)

### 2.2. Testing Methods

#### 2.2.1. Compressive Strength

The compressive strength of hardened SS pastes in the absence and presence of THEED was studied. The selected concentrations of THEED were 0 ppm, 500 ppm, 1000 ppm, and 2000 ppm, and the mass ratio of THEED solution to SS was kept at 0.35. The mixing of SS pastes was carried out according to “Test methods for water requirement of normal consistency, setting time and soundness of the portland cement” (GB/T 1346-2011). Steel slag and THEED solutions were mixed well in a 2.5 L stirring mixer for about 5 min at 125 rpm. The well-mixed steel slag paste was cast in molds with a size of 20 × 20 × 20 mm^3^, after which the specimens were put into a standard curing room for 3 days and then demolded. The compressive strength tests were conducted at 3 d, 7 d, and 28 d, and the average compressive strengths of the six specimens were calculated.

#### 2.2.2. Isothermal Calorimetry

Three g of steel slag was placed in an ampoule, and a specified concentration of THEED solution was injected into a syringe at a water–steel slag ratio of 0.35. Then the ampoule and syringe were assembled into a calorimetric channel. After checking the thermal equilibrium of the instrument, the THEED solution was injected into the ampoule and stirring was started; the stirring mode was internal stirring at a speed of 120 rpm for 2 min. The data were recorded from the moment of water injection, and the exotherm of hydration of the steel slag was measured continuously for 72 h at 25 ± 0.02 °C.

#### 2.2.3. XRD and TG Testing

The specimens with measured compressive strength were smashed into pieces and then immersed in isopropanol solution to terminate hydration for 48 h. After that, the pieces were dried in a vacuum oven at 40 °C for 48 h. The pieces were then ground into powders which passed through a 74 μm (200 mesh) sieve. A portion of the powder sample was used for XRD measurement with a Bruker D8 Advance diffractometer (Bruker, Germany) with a scanning speed of 5°/min from 5° to 50°. In addition, thermogravimetry (TG) measurement was conducted on about 15 mg of the powder sample using a NETZSCH STA 449F3 (NETZSCH, Selb, Germany) with a temperature range between 40° and 1000 °C under N_2_ atmosphere, at a temperature rise rate of 10 °C/min.

#### 2.2.4. Pore Structure Measurement

A mercury intrusion porosimetry (MIP) measurement was conducted on the sample pieces hydrated for 28 d using an Autopore IV 9510 tester (Norcross, GA, USA). Pore sizes were tested in the range from 5 nm to 340 μm. The relationship between pressure and pore size was calculated from the Washburn equation.

#### 2.2.5. Morphology Observation 

Scanning electron microscopy (FE-STEM SU9000, Hitachi, Japan) was used to characterize the microstructure of the sample pieces hydrated for 3 d. In this way, the effects of THEED on the hydration of SS at an early age was investigated.

#### 2.2.6. Elemental Concentrations of the Aqueous Phase of the SS Pastes

To investigate the effect of THEED on the early hydration of SS, two THEED concentrations of 0 ppm and 2000 ppm were selected. The SS was put into the PTFE beakers with the THEED solution, with the liquid to SS ratio selected as 5. When the hydration time reached 5 min, 1 h, 8 h, 16 h, and 24 h, the solution was filtered out using a 0.22 µm filter film, and the concentrations of the elements [Ca], [Al], [Si], and [Fe] were measured using ICP-AES (Optima 7000DV, Agilent, Santa Clara, CA, USA).

## 3. Results and Discussion

### 3.1. Compressive Strength

**Figure 1:**
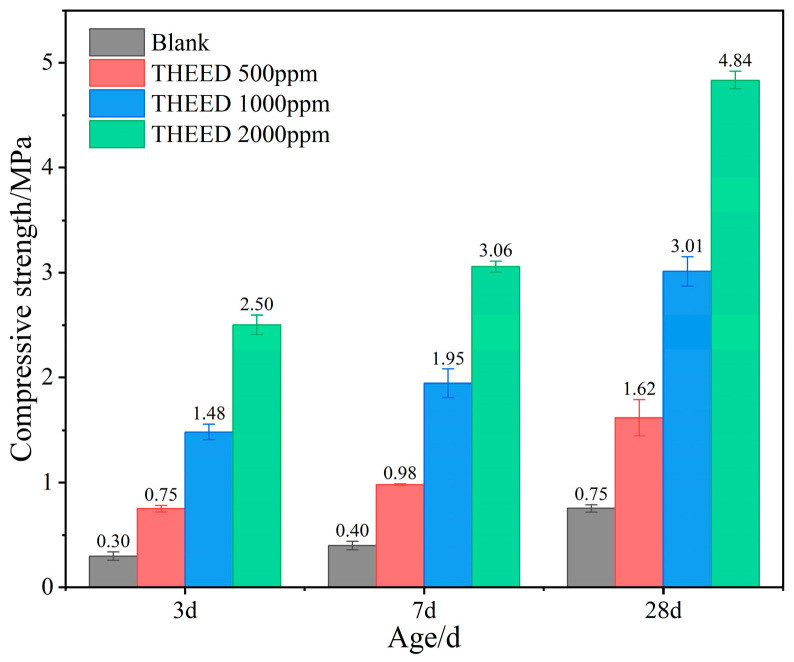
Compressive strength of hssp (W/C = 0.35) in absence and presence of THEED.

The compressive strengths of the hardened SS pastes (hssp) containing different concentrations of THEED are shown in Figure 1. The results show that the SS exhibit low hydraulic activity, indicated by the low compressive strength of the hssp before 28 d. It is interesting to find that THEED additions improved the compressive strength of the hssp significantly. The hssp with 2000 ppm THEED exhibits a 3 d compressive strength value of 2.5 MPa, while the strength of the blank paste is only 0.30 MPa. From 3 d to 28 d, the compressive strength of the hssp with and without 2000 ppm THEED increase by 2.34 MPa and 0.45 MPa, respectively. These results suggest THEED additions promote the strength development of the hssp, which is in agreement with the results of the hssp in the presence of EDIPA [2,11,23]. The enhancement effect increases with the concentration of THEED. Typically, the strength development of a cementitious system is affected by hydration kinetics, pore structure and product morphology. Therefore, further investigations have been conducted on the hydration process and microstructure of a pure SS system in the absence and presence of THEED.

### 3.2. Hydration Exotherm of the Hydrating SS Pastes

**Figure 2:**
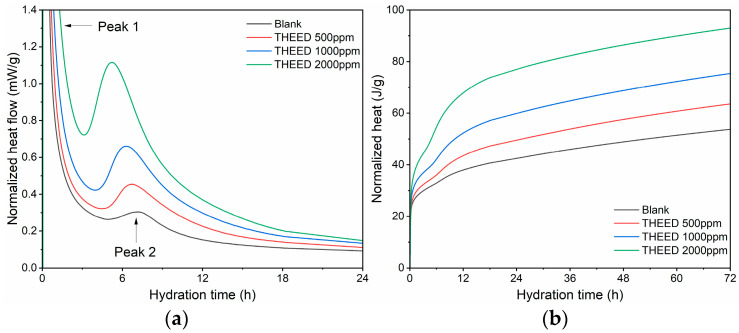
Hydration heat curves of SS (W/C = 0.35) with and without THEED. (**a**) Normalized heat flow; (**b**) Normalized heat.

**Figure 3:**
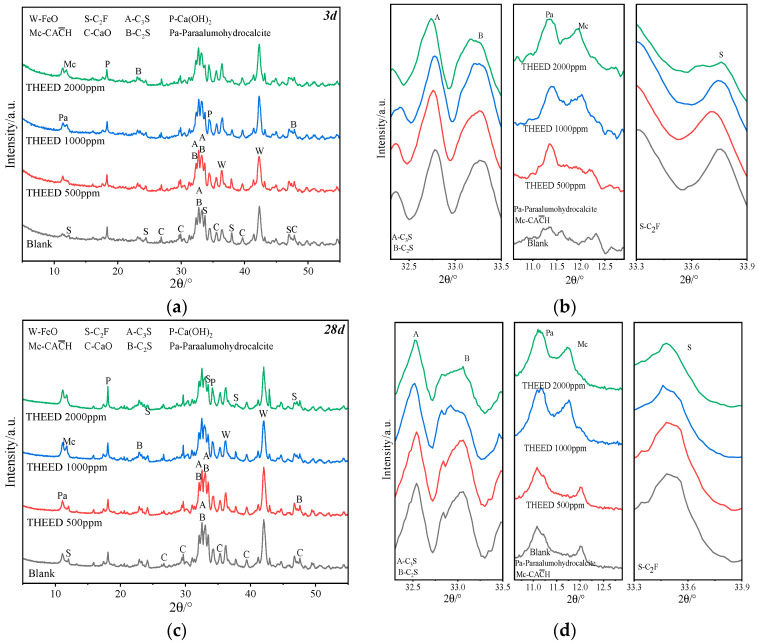
XRD pattern of hssp (W/C = 0.35) with and without THEED at 3 d (**a**) and 28 d (**c**), and characteristic peaks of Pa, Mc and C_2_F (**b**,**d**).

Figure 2 shows the heat flow and heat curves of hydrating SS pastes with and without THEED. It can be seen from Figure 2a that the hydration curve of SS includes two exothermic peaks, which is similar to that of cement. The first exothermic peak mainly results from the wetting of the minerals and the fast hydration of the aluminates, including C_3_A and C_12_A_7_, in the absence of gypsum [3,24]. The second one is considered to be the hydration of C_2_F or silicates (C_3_S, C_2_S) [2,11,19]. This exothermic peak is weaker than that of cement due to the limited amount of silicates present in SS, and the low cementitious activity of these phases. The induction period occurs between the two exothermic peaks. In the presence of THEED, a shorter duration of induction period and a higher intensity of the second exothermic peak of the hydrating SS pastes were observed, as seen in Figure 3a. These phenomena suggest that THEED additions accelerate the early hydration of SS. This effect becomes more prominent when a higher concentration of THEED is added into the SS pastes. Therefore, the cumulative heat flow of the SS pastes with THEED was higher than that of the blank SS pastes before 72 h, as presented in Figure 3b. The results are consistent with the compressive strength of the hssp at 3 d with and without THEED, depending on the fact that stronger cumulative heat release values correspond to higher compressive strength.

### 3.3. XRD of Hydrating SS Pastes

**Figure 4:**
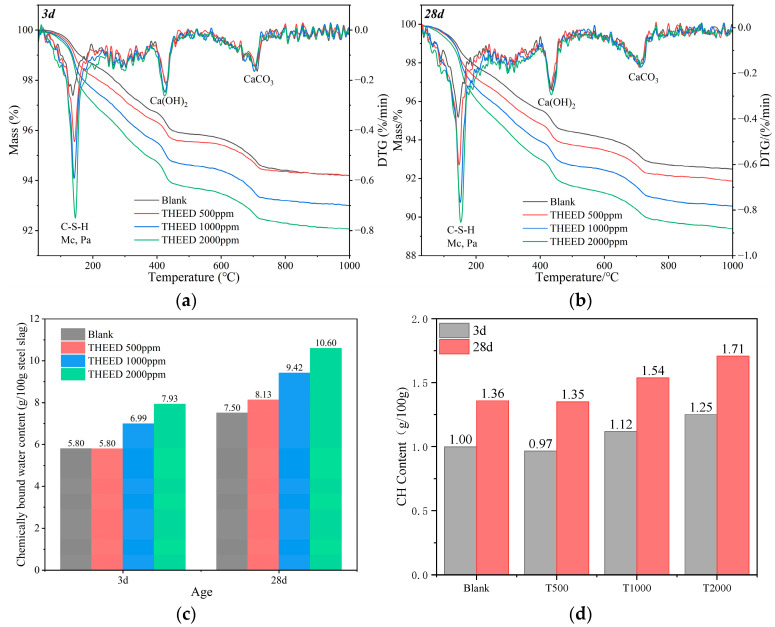
TG/DTG curve and CBW content of SS pastes (W/C = 0.35) at 3 d and 28 d in the presence of THEED. (**a**) 3 d; (**b**) 28 d; (**c**) CBW content; (**d**) CH content.

Hydration of SS is usually accompanied by changes in the mineral phase content. Figure 3a,c shows the XRD pattern of hydrating SS pastes in the presence of THEED at 3 d and 28 d, respectively. As seen in Figure 3, the main minerals in the SS pastes include aluminates (C_3_A, PDF-38-1429; C_12_A_7_, PDF-48-1882), while ferrates (C_2_F, PDF 38-0408) and silicates (C_3_S, PDF-16-0406; C_2_S, PDF-33-0302) are present in the hydrating SS pastes. Figure 3b,d depicts the characteristic peaks of paraalumohydrocalcite (Pa), monocarboaluminate (Mc, PDF-41-0219), and C_2_F (c) in the hydrating SS pastes at 3 d and 28 d, as observed by Chang et al. [2,11] It can be seen that the main hydration products of the aluminate phase at 3 d and 28 d in the presence of calcium carbonate are Pa and Mc, rather than Aft, due to the limited sulfate content in SS. It can be seen in Figure 3b,d that the intensity of the diffraction peaks of Pa and Mc in the hssp with THEED is higher than that of the blank SS pastes. This suggests that THEED promotes the formation of Pa and Mc, which may be a result of THEED promoting the hydration of aluminates. Moreover, the accelerating effect of THEED on Pa and Mc formation enhances with the increase of THEED concentration. It can also be noticed in Figure 3b,d that THEED also promoted the hydration of C_2_F at 3 d. The diffraction intensity of C_2_F decreases with the increase of THEED concentration, indicating that a higher concentration of THEED promotes the hydration of C_2_F more efficiently. It is worth mentioning that C_2_F hydration facilitates the formation of Pa and Mc, which is one of the reasons for the increase in intensity of the Pa and Mc diffraction peaks. The characteristic peaks of portlandite (CH) from silicate hydration and the raw material of SS are shown in Figure 4. The early hydration of silicates seems to be delayed by THEED addition, as indicated from the decreased intensity of the diffraction peak of CH. From 3 d to 28 d, the silicates hydrate to some extent as the intensity of the diffraction peak of CH increases. It was interesting to find that THEED additions lead to more crystal CH formation. However, the intensity of the diffraction peak of FeO remains unchanged in the SS pastes with different concentrations of THEED and different curing times, suggesting that little FeO participates in the hydration reaction. For these considerations, it is proposed that the second exothermic peak presented in Figure 2a mainly results from the hydration of C_2_F. Therefore, accelerating the hydration of the aluminate phases and C_2_F may be responsible for the enhancement of THEED in early strengths, and the acceleration of hydration of the silicates results in a higher strength at 28 d.

### 3.4. TG/DTG Analysis of Hydrating SS Pastes

TG/DTG curves were employed to reflect the influence of THEED on the hydration degree of the SS, as shown in Figure 4a,b. It can be seen that the TG curves of the hssp depict three mass loss stages, corresponding to the three mass loss peaks of the DTG. The first mass loss peak appears in the temperature range from 50~250 °C, which is a result of the dehydration of C-S-H, Pa, and Mc [25]. The second mass loss peak is shown in 400–500 °C, which is a result of the decomposition of Ca(OH)_2_ [26]. The third mass loss peak is observed in 600–800 °C, which can be attributed to the decarbonization of CaCO_3_ [27].

In the presence of THEED, the intensity of the first peak is significantly increased because of the fact that THEED promotes the early hydration of aluminates, silicates, and C_2_F, as confirmed by the XRD results. In addition, the CH and CaCO_3_ contents of the hardened SS pastes were calculated to determine the effect of THEED on the hydration of silicates. Results show that 1.64 g and 2.20 g CH per 100 g SS were present in the hardened SS pastes at 3 d and 28 d, respectively. The increase in CH content contributes to the further hydration of silicates. THEED additions have no obvious effect on the CH content of the hardened SS pastes at 3 d, but increase the CH content at 28 d from 2.20 g to 3.22 g CH per 100 g SS with a THEED concentration of 2000 ppm. This phenomenon suggests the hydration of silicates from 3 d to 28 d is accelerated by using THEED. The results also show 1.38 g and 1.53 g CaCO_3_ per 100 g SS in the hssp are detected at the age of 3 d and 28 d, respectively. It is found that THEED additions decrease the CaCO_3_ content slightly, which results from the accelerating hydration of aluminates and C_2_F to generate Pa and Mc, as indicated by the XRD patterns presented in Figure 3. Therefore, the hydration degrees of SS at 3 d and 28 d are increased by THEED, as indicated by the chemical bond water content (CBWC) results of the hssp presented in Figure 4c.

### 3.5. Pore Structure of Hydrating SS Pastes

**Figure 5:**
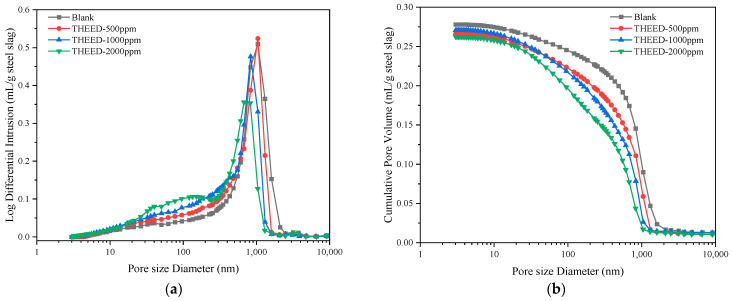
Pore size distribution of the hardened SS pastes (W/C = 0.35) with and without THEED at 28 d. (**a**) Log differential intrusion; (**b**) Cumulative pore volume.

**Figure 6:**
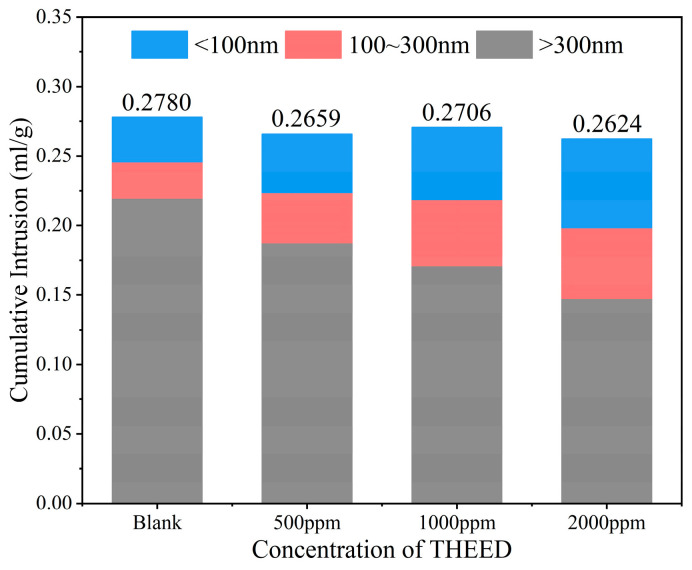
Pore structure of the hardened SS pastes (W/C = 0.35) with and without THEED at 28 d.

In the hydrating SS pastes, the dissolution of SS and precipitation of products occurred with the consumption of water [6,28]. Pore structure of the hssp was determined to reflect the hydration degree of SS, which determines the strength development [29,30]. The pore size distribution and cumulative pore volumes of the hssp at 28 d were determined via MIP, and the results are presented in Figure 5. The blank SS paste exhibits an average pore diameter of 148.8 nm and a total porosity of 46.4%. A distinguished pore size distribution peak appears at around 800 nm. In the presence of THEED, the position of the peak shifts to the left, suggesting the pore structure of the hssp is refined by THEED. The cumulative pore volume is reduced by THEED, as observed in Figure 5b. To further study the influence of THEED on the pore structure of hssp, the pores were divided into three categories including pore diameter within 100 nm, pore diameter in the range from 100 to 300 nm, and pore diameter beyond 300 nm. The volumes of the three scopes are presented in Figure 6. Results show THEED additions tend to decrease the volume of the pores with a diameter greater than 300 nm. This change is related to the accelerated hydration of SS by THEED. The reduced volume of the pores with a diameter greater than 300 nm results in increasing the compressive strength of the hssp at 28 d. It can be seen that the accelerated formation of hydration products by THEED splits the large pores into smaller pores of smaller size. This phenomenon further suggests that THEED promotes hydration of SS to achieve a higher strength of the hssp, and the enhancement effect becomes stronger with increasing THEED concentration.

### 3.6. Morphologies of Hardened SS Pastes

**Figure 7:**
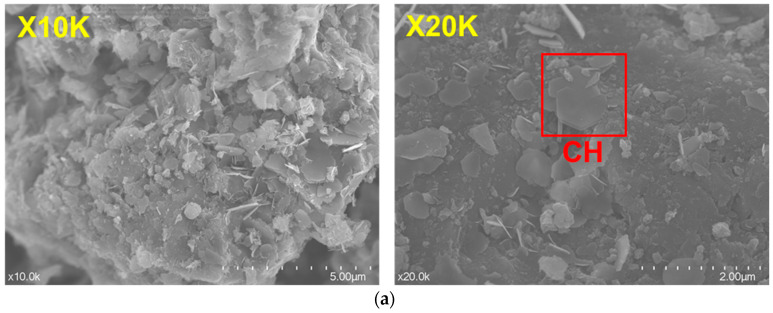

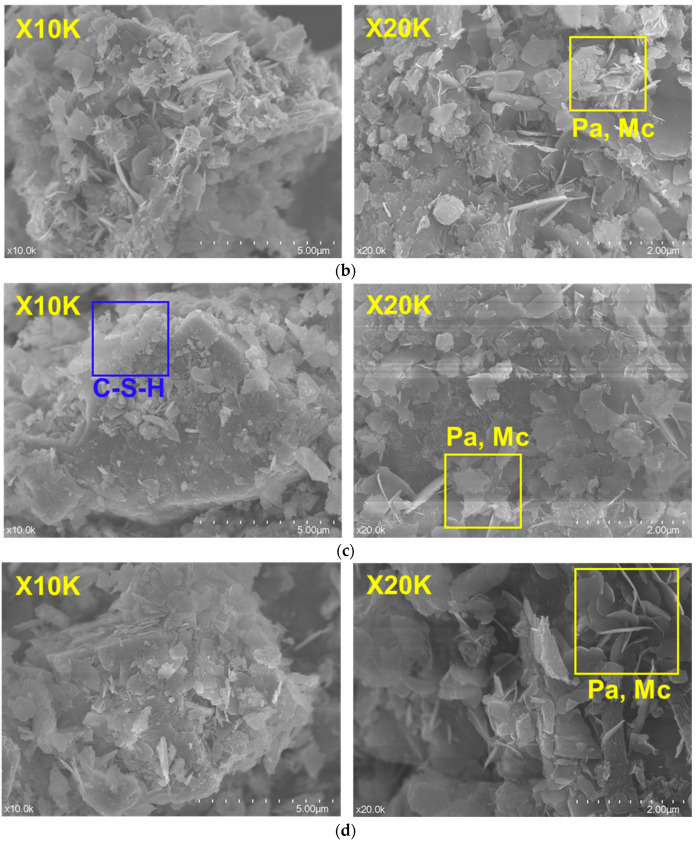
Morphologies of hydration products in hardened SS pastes at 3 d. (**a**) Blank; (**b**) THEED 500 ppm; (**c**) THEED 1000 ppm; and (**d**) THEED 2000 ppm.

Figure 7 shows the product morphologies in hssp hydrated for 3 d with various THEED concentrations. It is obvious that the hydration products with plate-like and flake shapes cover the surface of SS hydrated for 3 d. However, it is hard to identify the types of hydration products from the morphology of hydration products. Therefore, EDS was used for elemental analysis of the products. It confirmed that the plate-like products marked with red can be considered to be CH, and the flake-shaped products marked with yellow are Pa or Mc. Moreover, little amorphous C-S-H is observed in the hydrating SS pastes. From these observations, it is inferred that the silicates including C_3_S and C_2_S in the SS pastes have low hydration activity. Due to the fact they lack a sulfur carrier, aluminum and ferrite phases including C_3_A, C_12_A_7_ and C_2_F hydrate more rapidly. In the presence of CaCO_3_, the final hydration products of the aluminum and ferrite phases in the SS pastes are Pa and Mc, as confirmed in Figure 3. It was found that THEED additions have no obvious effect on the morphology of the hydration products. More hydration products can be observed in the hardened SS pastes with THEED, which is in agreement with results from the XRD and TG measurements.

### 3.7. Dissolution and Precipitate in Hydrating SS Pastes

**Figure 8:**
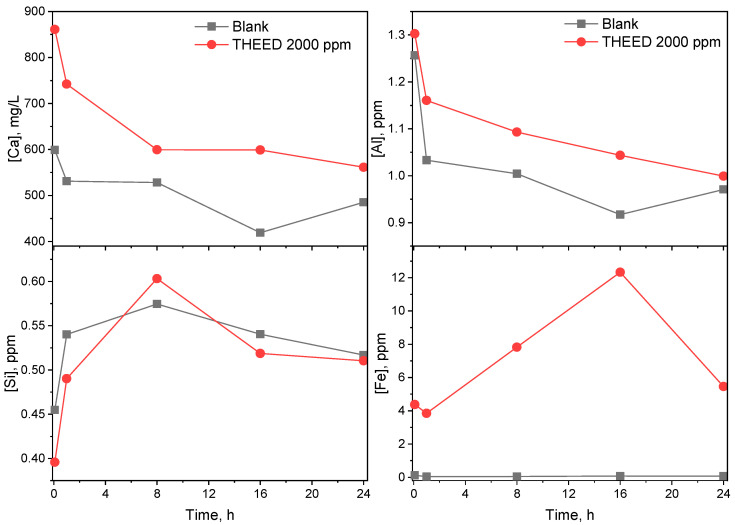
Concentration of [Ca], [Al], [Si], and [Fe] elements in aqueous phase of steel slag pastes in presence of THEED.

The dissolution of the SS was monitored in a diluted solution with a liquid to solid ratio of 5. The concentrations of the elements [Ca], [Al], [Si], and [Fe] in the aqueous phase of the SS paste hydrated for 5 min, 1 h, 8 h, 16 h and 24 h are presented in Figure 8. Results show that the SS suspensions with various concentrations of THEED are rich in [Ca] and lacking in [Al] and [Fe] before the first 24 h. In the blank SS paste, the concentrations of [Ca] and [Al] are 599 ppm and 1.26 ppm in the SS pastes after 5 min. Then the concentrations of [Ca] and [Al] tend to decrease with time due to the precipitation of products. In the presence of 2000 ppm THEED, the concentrations of [Ca] and [Al] are higher than that of the blank SS pastes. In particular, little [Fe] is detected in the blank SS pastes. Notably, the concentrations of [Fe] are increased by THEED addition. The higher concentration is considered a result of THEED promoting the dissolution of the aluminates and ferrite phases. After hydration for 16 h, the concentration of [Fe] decreases sharply in the SS pastes with THEED, suggesting the ferrite-containing products start to quickly precipitate. Moreover, SS pastes with a low concentration of [Si] are observed before 24 h. From the evolution of the concentrations of [Si], it is seen that THEED has little interference on the dissolution of silicates including C_3_S and C_2_S. Therefore, the impact of THEED on the liquid phase environment of the SS pastes is probably the reason for accelerating SS hydration.

### 3.8. Acceleration Mechanism of THEED

**Figure 9:**
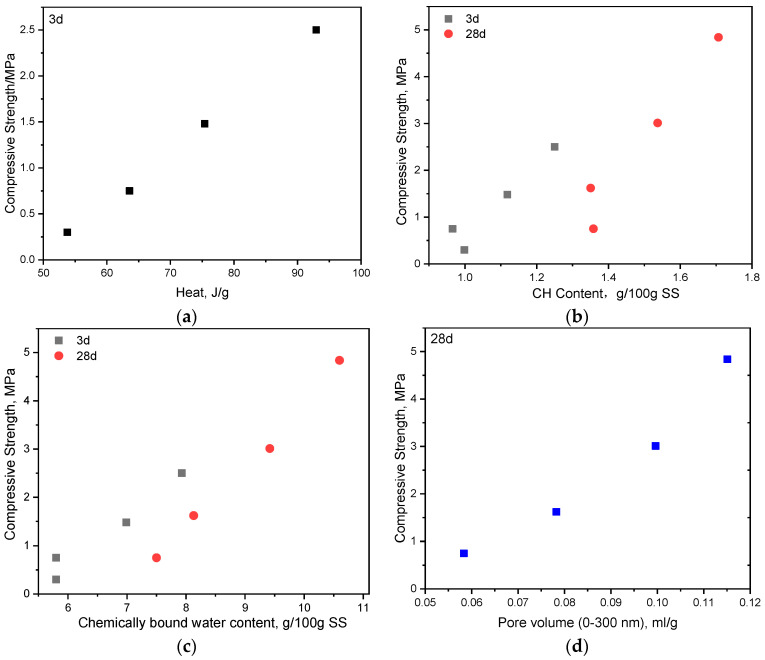
Relationships between compressive strength and hydration process parameters of SS. (**a**) Compressive strength vs. heat release; (**b**) Compressive strength vs. CH content; (**c**) Compressive strength vs. CBW content; (**d**) compressive strength vs. pore volume.

From the above results, it is found that the compressive strength of the hssp is enhanced by THEED, and the enhancement effect increases with the concentration of THEED. At a concentration of 2000 ppm, THEED increases the strength of the hssp by 733%, 665%, and 545% at 3 d, 7 d and 28 d, respectively. Through investigating the hydration process of the SS with and without THEED, it is confirmed that THEED additions accelerate the hydration of the aluminate and ferrate phases in SS pastes, resulting in the formation of Mc and Pa in the presence of CaCO_3_. A higher concentration of THEED leads to a more prominent effect. Also, the increased CH content of the hssp with THEED also suggests THEED accelerates the hydration of silicates (C_3_S and C_2_S) at 3 d and 28 d. In this way, the chemically bonded water content of the hssp is increased by THEED, and a more condensed structure of the hssp with THEED is obtained. To further underscore the mechanism of THEED, the relationships between compressive strength and the hydration process parameters of SS, including the cumulative heat released at 3 d; the CBW and CH content; and pore volumes with a diameter below 300 nm are established, as seen in Figure 9. It is found in Figure 9a that the 3 d strength of the hssp increases linearly with the cumulative heat released at 3 d, suggesting the hydration kinetics of SS determines the early strength development. However, the CH and CBW contents change irregularly with the compressive strength, suggesting the compressive strength is not determined by silicate hydration or aluminate/ferrite hydration individually. Figure 9d shows the enhancement of THEED additions decrease the pore volume (0–300 nm), resulting in increasing the strength. These changes are responsible for the increased compressive strength of the hssp with THEED.

To reveal the driving force of the hydration of SS in the presence of THEED, the concentrations of elements including [Ca], [Al], [Fe], and [Si] are measured via ICP. Results show that THEED significantly improves the concentration of [Ca], [Al], and [Fe] in the steel slag paste, indicating THEED accelerates the dissolution of clinkers in SS. The solubilizing effect of THEED is supposed to be related to its chelating behavior with metal ions, due to the fact that the chemicals with the hydroxyl and amino groups as TEA exhibit the ability to chelate with metal ions in cementitious pastes. The chelating solubilization effect was also proposed in our previous research work [11]. This effect is believed to offer the driving force for promoting the hydration of steel slag. In SS pastes, THEED chelates with metal ions to form complexes. Taking C_2_F as an example, the dissolution of C_2_F and the chelating process of THEED are shown in Equations (1)–(3) as follows:

(1) $$2\mathrm{CaO}\cdot {\mathrm{Fe}}_{2}O_{3}+H_{2}O+{2\mathrm{OH}}^{-}\to {2\mathrm{Ca}}^{2+}+{2\mathrm{Fe}(\mathrm{OH})}_{4}^{-}$$

(2) $$n\mathrm{THEED}+{\mathrm{Ca}}^{2+}\to {[\mathrm{nTHEED}\cdot \mathrm{Ca}]}^{2+}$$

(3) $$\mathrm{nTHEED}+{\mathrm{Fe}(\mathrm{OH})}_{4}^{-}\to {[\mathrm{nTHEED}\cdot {\mathrm{Fe}(\mathrm{OH})}_{4}]}^{-}$$

According to Le Chatelier’s principle, the chelating of THEED with Ca^2+^ and ${\mathrm{Fe}(\mathrm{OH})}_{4}^{-}$ reduces the concentration of free Ca^2+^ and ${\mathrm{Fe}(\mathrm{OH})}_{4}^{-}$, thus promoting the dissolution of C_2_F [2,11]. In this way, the precipitation of a hydration product is accelerated. For aluminate hydration, C_3_A and C_12_A_7_ undergo a similar chelating effect of THEED to promote their hydration. This effect could explain the reason for accelerating the silicate hydration by THEED.

In summary, the mechanism of THEED in the hydration process of steel slag can be expressed as follows: THEED promotes the dissolution of mineral phase ions in steel slag via chelating to achieve higher concentrations of metal ions, promoting the precipitation of hydration products.

## 4. Conclusions

The effect of THEED on the strength development of a pure steel slag system was investigated. From hydration kinetics, pore structure and microstructure analyses of the hydrating SS pastes with and without THEED, the working mechanism of THEED is proposed. Based on the above results, the following conclusions can be drawn:

(1) THEED additions significantly increase the compressive strength of hardened steel slag paste in the early (3 d) and late (28 d) stages. The enhancement effect increases with the dosage of THEED. At a concentration of 2000 ppm, THEED increases the compressive strength of hardened SS pastes by 733%, 665%, 545% at 3 d, 7 d, and 28 d, respectively.
(2) THEED additions improve the hydration degree of SS by accelerating the hydration of the aluminum phase (C_3_A, C_12_A_7_) and the ferrite phase (C_2_F) to form Mc and Pa in the presence of CaCO_3_. Also, the silicate hydration is increased by THEED. In this way, THEED refines the pore structure of the hardened steel slag paste by increasing the pore volume with a diameter below 300 nm to achieve an enhancement in compressive strength.
(3) A working mechanism of THEED for the enhancement is proposed. The chelating solubilization effect of THEED is believed to offer the driving force for promoting the hydration of SS. The dissolution of SS is accelerated by THEED to promote the precipitation of hydration products.

## Figures and Tables

**Table 1:** Chemical composition of SS.

Chemical Composition	CaO	SiO_2_	Al_2_O_3_	Fe_2_O_3_	MgO	SO_3_	Cl	LOI	Others
wt%	39.58	12.41	4.05	25.57	5.54	0.35	0.12	1.85	10.53

**Table 2:** Mineral composition of SS.

Mineral Composition	C_3_S	C_2_S	C_3_A	C_12_A_7_	C_2_F	FeO	Ca(OH)_2_	CaCO_3_
wt%	6.48	27.78	7.95	0.57	9.78	12.32	0.64	0.88

## Data Availability

Data are contained within the article.
